# A New Chitosan-Modified Paper-Based SERS Glucose Sensor with Enhanced Reproducibility, Stability, and Sensitivity for Non-Enzymatic Label-Free Detection

**DOI:** 10.3390/bios15030153

**Published:** 2025-03-01

**Authors:** Rashida Akter, Toeun Kim, Jong Seob Choi, Hongki Kim

**Affiliations:** 1 Department of Chemistry, Kongju National University, Gongju-si 32588, Republic of Korea; rashidaakter@kongju.ac.kr (R.A.); toeungim@gmail.com (T.K.); 2 Division of Advanced Materials Engineering, Kongju National University, Budaedong 275, Seobuk-gu, Cheonan-si 31080, Republic of Korea; choijongseob@kongju.ac.kr

**Keywords:** surface-enhanced Raman scattering, cellulose paper, chitosan, silver nanoparticles, 4-mercaptophenyl boronic acid, glucose sensor, human blood serum sample

## Abstract

We have fabricated a new highly reproducible, stable, and sensitive cellulose paper-based Surfaced-enhanced Raman scattering (SERS) sensor substrate for non-enzymatic label-free glucose detection. To enhance reproducibility, stability, and sensitivity, the cellulose paper (CP) substrate has been modified with a naturally derived biocompatible polymer, chitosan (CS), followed by depositing enormous amount of plasmonic silver nanoparticles (AgNPs) on CP/CS and finally forming a self-assembling monolayer of 4-mercaptophenyl boronic acid (MPBA) on CP/CS/AgNPs (CP/CS/AgNPs/MPBA). The SERS sensor substrate is characterized by scanning electron microscopy (SEM), energy dispersive X-ray (EDX), Fourier transform infrared (FT-IR), and X-ray diffraction (XRD) spectroscopy techniques. The glucose sensing is achieved by monitoring the SERS intensity of C-S and B-O stretching vibrations at 1072 cm^−1^ in MPBA, which is gradually increased with increasing concentration of glucose due to the increasing orientation change of MPBA on AgNPs. The results show that the proposed glucose paper-based SERS sensor exhibits a high analytical enhancement factor (AEF) (3.4 × 10^7^), enhanced reproducibility (<7%), improved stability (>5 weeks), excellent selectivity towards other metabolic compounds, and high sensitivity with a limit of detection (LOD) of 0.74 mM and a linear dynamic range between 1.0 and 7.0 mM. The practical application of this SERS sensor is examined in real spiked and non-spiked human blood serum samples for the detection of glucose, and satisfactory recovery results have been obtained, demonstrating the potentiality of the present paper-based SERS sensor for non-enzymatic label-free glucose detection in real biological samples.

## 1. Introduction

Diabetes mellitus (DM) is a chronic metabolic disease associated with high glucose level, which is considered to be a serious threat to human health and life [1]. The physiological level of glucose in human blood is 3.9~6.1 mM (70~110 mg/dL). If the glucose concentration is found to be higher than this physiological level, diabetes (DB) complications can occur, which are directly linked to kidney failure, heart disease, stroke, blindness, nerve diseases, and so on [2]. As such, glucose detection is essential for diabetes control and monitoring. Therefore, various analytical detection methods such as high-performance liquid chromatography (HPLC) [3], colorimetric [4,5,6,7,8], electrochemical [9,10,11], fluorescence [12,13,14], mass spectrometry [15], chemiluninescence [16], and surface-enhanced Raman scattering (SERS) [17,18,19,20,21] have been studied. Among the various glucose detection methods, SERS has shown significant advantages due to its fast, highly sensitive, selective, non-destructive, and molecular fingerprinting [22,23,24].

SERS is a plasmonic phenomenon that arises due to the amplification of scattering on metallic nanoparticles surface. This amplification occurs due to the localized surface plasmon resonance (SPR), a collective oscillation of conductive band electrons produced by the interaction between plasmonic nanomaterials and light at a specific wavelength [25], which gives a strong enhancement of the Raman signals. This strong enhancement is largely attributed to the existence of highly excited regions (hot spots). At these hot spots, the electric field becomes highly augmented during the interaction of the incident light with the small nm gaps of plasmonic nanostructures [26]. The SERS enhancement is related to two mechanisms: the electromagnetic and chemical effect [27,28,29,30,31]. The electromagnetic effect occurs due to the excitation of localized surface plasmon resonance (LSPR), when a molecule is adsorbed on a laser-irradiated noble metal nanostructured surface. The chemical enhancement occurs due to the direct charge transfer interactions between a molecule and the metal surface that is chemically adsorbed on. In both mechanisms, SERS substrate plays a key role for signal enhancements.

The SERS enhancement of SERS substrates can be evaluated via the enhancement factor (EF) or analytical enhancement factor (AEF), which are largely determined by the structure and numbers of hot spots [30]. The EF or AEF of typical SERS substrates is in a range between 10^4^ and 10^7^, although some SERS substrates that are precisely fabricated or assembled to have small nm gaps on flat substrates exhibit high EF ranging from 10^7^–10^14^ with strong SERS reporters [32]. Lithography and chemical assembly-based SERS substrates prepared on flat substrates like silicon [33], glass [34], and alumina [35] can provide higher EFs. However, the fabrication of solid SERS substrates is neither cost-effective nor easily available. Therefore, the fabrication of cheap and easily prepared SERS substrate is highly desirable, especially for the detection of glucose.

Paper-based chemical and biological assays have attracted lots of interest due to their cost-effectiveness, ease of use, portability, flexibility, passive liquid transportation, chemical and biological compatibility, etc. [36]. Various paper-based analytical approaches have been developed, combining techniques such as ELISA [37], electrochemistry [38], fluorescence [39], chemiluminescence [40], and Raman spectroscopy [41]. Among these analytical techniques, Raman spectroscopy [41] has been promising for finger-print identification of chemical composition. Recently, we have developed a paper-based SERS biosensor for highly sensitive food spoilage detection [42]. Paper-based SERS substrates could have advantages over those well-defined SERS substrates in terms of cost, eco-friendly disposability, flexibility, and integration with microfluidic devices and sensors [43]. For example, various paper-based microfluidic or non-microfluidic SERS sensors for trace amounts of phenolic and thiolic compounds, environmental pollutants, food spoilage, and biomolecules have been reported [44,45,46,47,48,49,50,51,52,53,54,55,56,57,58,59,60]. The SERS-based non-enzymatic and enzymatic glucose detection on solid metal nano-surfaces has been extensively studied and recently reviewed [61]; however, paper-based non-microfluidic glucose SERS sensors have not been yet studied. The main drawback of the non-microfluidic paper-based SERS sensor is its low sensitivity, reproducibility, and stability. In order to improve the sensitivity, reproducibility, and stability, the paper-based SERS substrate needs to be coated with a natural biocompatible polymer which has available functional sites for nanomaterials attachments.

In the present study, chitosan (CS) [62,63], a biocompatible and biodegradable cationic polymer with amine functional sites has been coated on a cellulose paper (CP) [64] surface for reproducibility, sensitivity, and stability enhancement. CS has been stably coated on the CP surface through the charge interaction between the negative charges of CP and positive charges of CS. Plasmonic AgNPs have then been uniformly deposited on the CP/CS through the N-Ag bonding, thus providing a stable and reproducible paper-based SERS sensor substrate. Instead of using an enzyme, we used a synthetic molecular receptor, 4-mercaptophenyl boronic acid [65], for the selective detection of glucose. The CP/CS/AgNPs-based SERS platform has been characterized by using scanning electron microscopy (SEM), energy dispersive X-ray (EDX) spectroscopy, Fourier transform infrared (FT-IR), and X-ray diffraction (XRD) spectroscopy techniques. Various experimental conditions such as CS and MPBA concentrations and the pH of the detection media have been optimized, and the CP/CS/AgNPs/MPBA-based SERS sensor has been successfully used for glucose detection in phosphate buffer saline (PBS) solutions and in real blood serum samples. The results show that the chitosan modification of the cellulose paper enhanced the reproducibility, stability, and sensitivity of the SERS-based glucose sensing in real human serum samples.

## 2. Materials and Methods

### 2.1. Materials

Silver nitrate (AgNO_3_, 99.999%), sodium borohydride (NaBH_4_, 98.0%), chitosan (Low molecular weight), 4-mercaptophenylboronic acid (MPBA, 90%), glucose (C_6_H_12_O_6_, 99.5%), uric acid (C_5_H_4_N_4_O_3_, 99%), acetic acid (CH_3_CO_2_H, 99.7%), ascorbic acid (C_6_H_8_O_6_, 99%), dopamine hydrochloride ((HO)_2_C_6_H_3_CH_2_NH_2_HCl, 98%), acetaminophen, lactic acid, and human serum sample were purchased from Sigma Aldrich Co. (St. Lous, MI, USA) Cellulose paper was purchased from Whatman Co. (Marlborough, MA, USA).

### 2.2. Apparatus

Scanning electron microscope (SEM) images were measured using high resolution field emission scanning electron microscope (HR FE-SEM, model MIRA3-LMH, Tescan, Brno, Czech Republic). EDX measurements were carried out with a HR FE-SEM, model MIRA3-LMH, Tescan. The X-ray diffraction (XRD) analysis was carried out using an X-ray diffractometer (model MiniFlex 600, model Rigaku, Tokyo, Japan). Fourier transform infra-red (FT-IR) experiments were performed using a FT-IR spectrometer (IRAffinity-1, SHIMADZU, Kyoto, Japan). All SERS spectra were monitored using a custom Raman read-out system for large-area scanning (Micro Raman, UniNanoTech, Gyeonggi-do, Republic of Korea). A 632.9 nm laser was used as the excitation source. The acquisition time was 10 s using a 10× objective lens.

### 2.3. Fabrication of CP/CS/AgNPs/MPBA-Based SERS Sensor Substrate

**Figure 1:**
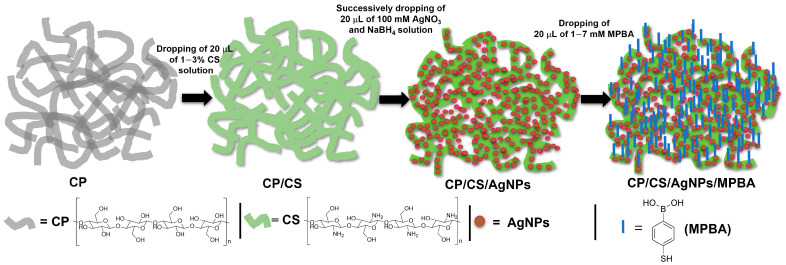
Schematic illustration of the fabrication of CP/CS/AgNPs/MPBA-based SERS sensor substrate. The chemical structures of CP, CS, and MPBA are shown below the scheme.

The CP was first cleaned with 10% HNO_3_ and 10% NaOH and dried at room temperature. After cleaning and drying, the CP was cut into a small piece of 1/1 cm size. To make a chitosan modified CP substrate (CP/CS), 20 μL of 1~3% chitosan solution was dropped onto the CP surface to cover all exposed area and kept at room temperature for 20 min to dry. Then, 20 μL of 100 mM AgNO_3_ solution were dropped onto the CP/CS surface followed by dropping 20 μL of 100 mM sodium borohydride solution and kept for 20 min to make AgNPs. The CP/CS/AgNPs substrate was washed with distilled water and dried. The color of the CP/CS surface dramatically changed from white to dark brown, indicating the formation of AgNPs was caused by the reducing action of borohydride from Ag^+^ to Ag^0^. Then, 20 μL of 1~7 mM MPBA solutions at pHs 3–10 were dropped on the dried CP/CS/AgNPs surface and kept for 1 h to complete the fabrication of the CP/CS/AgNPs/MPBA substrate. Following this, 20 μL of 1~7 mM concentration of glucose solutions at pH 7.4 were dropped on the CP/CS/AgNPs/MPBA substrate and kept for 10 min at room temperature for glucose binding. After washing with distilled water and drying in room temperature, the SERS spectra were monitored. The schematic illustration of the fabrication of the CP/CS/AgNPs/MPBA-based SERS sensor substrate is shown in Figure 1.

## 3. Results

### 3.1. Preparation of CP/CS/AgNPs/MPBA SERS Sensor Substrate

In order to prepare the chitosan (CS) -modified CP, various concentrations (1–5%) of CS solutions were dropped on the cleaned and dried CP surfaces. CP backbone surfaces contain enormous numbers of OH groups, whereas chitosan has both OH and NH_2_ groups. The CS was coated on the CP surface through the electrostatic interaction, formation of hydrogen bonds, and van der Waals forces. The CS coated CP (CP/CS) surfaces have both OH and NH_2_ groups. After dropping the Ag^+^ ion solution on the CP/CS surfaces, the negatively charged OH groups strongly attracted the positively charged Ag^+^ ion through electrostatic interaction, which lead to much more adsorption of Ag^+^ ion. After the borohydride reduction, the adsorbed Ag^+^ ions were reduced to AgNPs, which were in situ covalently bonded to the NH_2_ groups (CP/CS/AgNPs) surfaces. The amount of the deposited AgNPs on the CP/CS surface strongly depended on the amount of CS adsorption on the CS surfaces. The color of the CP/CS surfaces rapidly changed to a dark brown color after borohydride reduction, indicating the formation of AgNPs on the CP/CS surfaces. Both the nucleation and nanoparticle formation of Ag ions simultaneously occurred at the CP/CS surface. The selective glucose-binding receptor MPBA then self-assembled on the CP/CS/AgNPs surfaces through the interaction between AgNPs and SH groups of MPBA, yielding a stable and robust SERS sensor platform for glucose detection.

### 3.2. Characterization of CP/CS/AgNPs/MPBA-Based SERS Substrate

**Figure 2:**
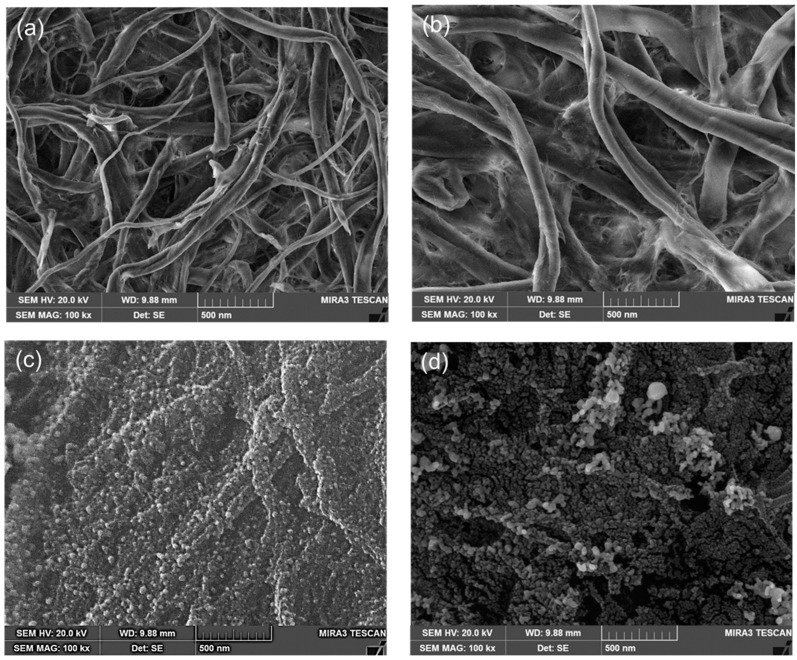
SEM images of the (**a**) CP-, (**b**) CP/CS-, (**c**) CP/CS/AgNPs-, and (**d**) CP/CS/AgNPs/MPBA-based sensor surfaces.

The CP/CS/AgNPs/MPBA surfaces were characterized using SEM, EDX, and XRD techniques. Figure 2 shows the SEM images of the CP, CP/CS, CP/CS/AgNPs, and CP/CS/AgNPs/MPBA surfaces. Both nano- and micro-sized fibers with porous morphology were observed in the SEM image of CP (Figure 2a). The increased widths of the fibers were observed after CS modification (Figure 2b) and the CP fibers were found to be more aggregated in the CP/CS surface. The porous morphology of CP surface was almost covered by the CS layer in the CP/CS. In the CP/CS AgNPs (Figure 2c), the AgNPs were clearly seen on the CP/CS surfaces. The AgNPs were uniformly and homogeneously deposited on the CP/CS surfaces with some aggregated cluster. The particle sizes of the AgNPs were approximately 10–30 nm, although some smaller particles were also observed. A Gaussian curve for the particle size distribution of AgNPs is plotted and presented in the Supplementary Materials as Figure S1. The average particle size was determined to be 26.74 ± 6.62. These results clearly showed that AgNPs strongly binds to the CP/CS surface through the interaction between the Ag and NH_2_ groups in CS. After MPBA binding (Figure 2d), the particle sizes of the AgNPs increased, and the uniform and homogeneous particle size distribution with some island-like morphology was observed. Additionally, some small rod-shaped AgNPs were observed due to the attachment of several single AgNPs upon MPBA binding.

**Figure 3:**
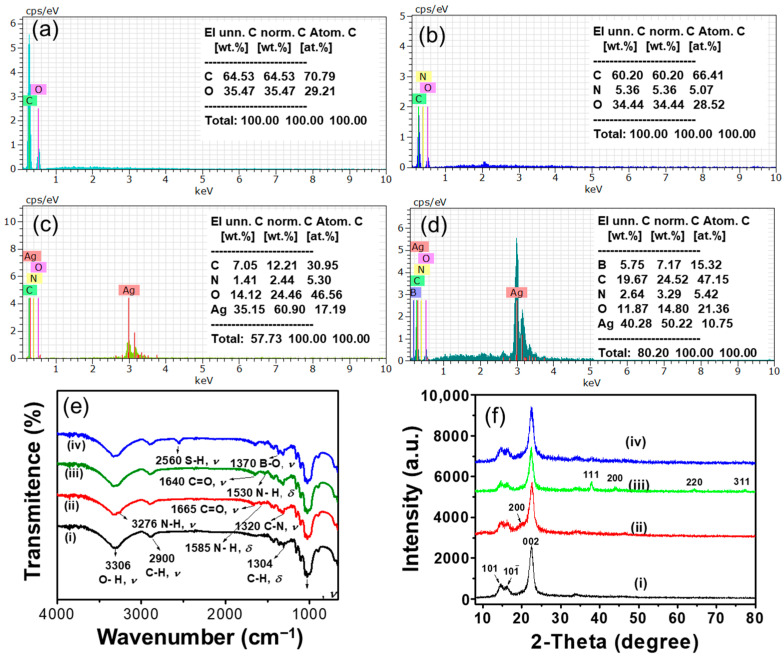
EDX spectra of (**a**) CP, (**b**) CP/CS, (**c**) CP/CS/AgNPs, and (**d**) CP/CS/AgNPs/MPBA surfaces. (**e**) FT-IR spectra and (**f**) XRD diffractograms of (i) CP, (ii) CP/CS, (iii) CP/CS/AgNPs, and (iv) CP/CS/AgNPs/MPBA-based sensor surfaces.

In order to confirm the SEM surface characterization, EDX analysis was carried out. Figure 3 showed the EDX spectra obtained for various surfaces. In the EDX spectrum of CP (Figure 3a), C and O peaks were observed. After CS modification on CP (Figure 3b), a new N peak was observed along with C and O peaks, indicating that the CS was attached on the CP surface. The EDX spectrum of CP/CS/AgNPs (Figure 3c) showed an additional Ag peak with C, O, and N peaks, showing the successful deposition of the AgNPs on the CP/CS surfaces. The presence of the B peak, along with C, O, N, and Ag peaks in the CP/CS/AgNPs/MPBA surfaces (Figure 3d), clearly indicates that the MPBA was successfully attached onto the CP/CS/AgNPs surfaces. The chemical analysis of the CP/CS, CP/CS/AgNPs, and CP/CS/AgNPs/MPBA surfaces revealed that the amount of N remained almost unchanged at about 5 w%, indicating the N binding sites in CP/CS were already saturated by AgNPs. The CP/CS/AgNPs/MPBA substrate was further characterized using FT-IR spectroscopy. Figure 3e showed the FT-IR spectra of individual CP, CP/CS, CP/CS/AgNPs, CP/CS/AgNPs/MPBA substrates. In the FT-IR spectrum of CP (Figure 3e(i)), three strong absorption bands at 3306, 2900, 1028 cm^−1^ were noted, which were attributed to O-H, C-H, and C-O stretching vibrations [66]. A weak absorption band at 1304 cm^−1^ was also observed, corresponding to the C-H bending vibration. In the spectrum of CP/CS (Figure 3e(ii), four absorbance bands were observed at 3276, 1665, 1585, and 1320 cm^−1^ along with other CP absorbance bands. These bands were attributed to the N-H stretching, C=O stretching, N-H bending, and C-N stretching vibrations in chitosan [67]. The presence of these absorbance bands in CP/CS indicates that CS was successfully modified on CP. The FT-IR spectra of CP/CS/AgNPs (Figure 3e(iii)) substrate showed all characteristic peaks of cellulose and chitosan. No new bands were observed for AgNPs; however, the C=O stretching and N-H bending peaks at 1665 and 1585 cm^−1^ in CP/CS shifted to the lower wavenumber (blue shift) and appeared at 1640 and 1530 cm^−1^. These blue shifts of C=O and N-H bands in CP/CS/AgNPs substrate were attributed to the interaction of nitrogen atom in primary amine and amide groups to AgNPs [67]. These results clearly prove the AgNPs were successfully deposited on CP/CS. The FT-IR spectrum of CP/CS/AgNPs/MPBA (Figure 3e(iv)) showed all the characteristic bands of CP and CS along with two additional bands at 2560 and 1370 cm^−1^, which are attributed to the S-H and B-O stretching vibrations [68]. The presence of B-O and S-H stretching bands in the CP/CS/AgNPs/MPBA substrate clearly confirm the MPBA attachment on the CP/CS/AgNPs through Ag-S interaction. In order to further confirm the AgNPs’ attachment on the CP/CS surface, the CP/CS surface, the CP, CP/CS, CP/CS/AgNPs, and CP/CS/AgNPs/MPBA surfaces were further characterized using the XRD technique. Figure 3f showed the XRD diffracrograms of CP, CP/CS, CP/CS/AgNPs, and CP/CS/AgNPs/MPBA. In the CP diffractogram, two secondary and one principal peaks at two theta angles (2θ) of around 15°, 16.5°, and 23° were observed, corresponding to (101), (101), and (002) crystal reflection planes, respectively, which are the typical patterns of native cellulose I allomorphs [66]. After CS modification, an additional broad small peak was observed at the 2θ of about 20°, corresponding to the (200) reflection plane [69], indicating the successful modification of CS on CP. The observation of the small broad peak of CS in CP/CS might be related to the overlapping of the CS (200) peak at 20° and CP (002) peak at 23°. To confirm this, we separately measured the XRDs of CS and CP/CS, and the diffractograms are presented in Figure S2. From the individual diffractogram of CP/CS and CS, it is obvious that the CP and CS peaks overlapped. Importantly, the crystallinity of CP did not decrease upon CS modification as the nature of the principal sharp and intense peak for 002 crystal plane at 23° in CP was not found to be broadened and decreased. After AgNPs deposition, the four distinct diffraction peaks in CP/CS/AgNPs at 38.1°, 44.1°, 64.5°, and 78.1° along with other peaks in CP and CS were observed. These peaks were assigned to the crystalline planes of (111), (200), (220), and (311), respectively, indicating the AgNPs are fcc-type unit cells. These Ag peaks were not observed in CP and CP/CS surfaces, indicating the presence of AgNPs on the CP/CS surfaces. The XRD diffractogram of the CP/CS/AgNPs/MPBA did not show any new peak. However, the sharp nature and high intensity of all Ag peaks changed to a broader type with decreased intensity, indicating MPBA was successfully attached on the CP/CS/AgNPs. These observations were also previously observed in the XRD analysis of MPBA-activated AuNPs surface [65].

### 3.3. Determination of Analytical Enhancement Factor of CP/CS/AgNPs Sensor Substrate

SERS enhancements of the CP/CS/AgNPs substrate were evaluated by determining the analytical enhancement factor (AEF) using rodamine 6G (R6G). The AEF value was determined with Raman band of R6G at 612 cm^−1^ using the following equation [70]:

AEF = (*I*_SERS_/*C*_SERS_)/(*I*_Raman_/*C*_Raman_) (1)

where *I*_SERS_ and *I*_Raman_ are the SERS intensity and the normal Raman intensity, and *C*_SERS_ and *C*_Raman_ are the concentrations of analyte in the SERS and control Raman experiments, respectively. Detailed AEF calculation and the SERS and Raman spectra of R6G are shown in Figure S3 in the Supporting Information. Based on the above equation, the AEF of the CP/CS/AgNPs was estimated to be 3.7 × 10^7^. This value is much higher than the AEF values of 6.55 × 10^6^ and 2.98 × 10^6^ obtained for PMHS-modified paper/SiO_2_/AgNCs and normal paper/SiO_2_/AgNCs substrates, respectively [71]. Our AEF value can also be compared to other EF values of 6.4 × 10^5^ for filter paper/AgNPs [47], 3.93 × 10^5^ for paper/AgNPs [50] and similar to the EF values of 2.8 × 10^7^ for filter paper/AgNPs [51], 2.1 × 10^7^ for filter paper/AgNPs [54], 2.0 × 10^7^ for paper/microfluidic paper/AgNPs [57], 1.0 × 10^7^ for cellulose paper/AgNPs [58], and 3.2 × 10^7^ for filter paper/AgNPs [59] substrates. These findings clearly demonstrate that the present CP/CS/AgNPs-based SERS glucose sensor substrate exhibits excellent SERS enhancement compare to other SERS substrates.

### 3.4. SERS Responses of CP/CS/AgNPs/MPBA-Based Sensor Substrate

**Figure 4:**
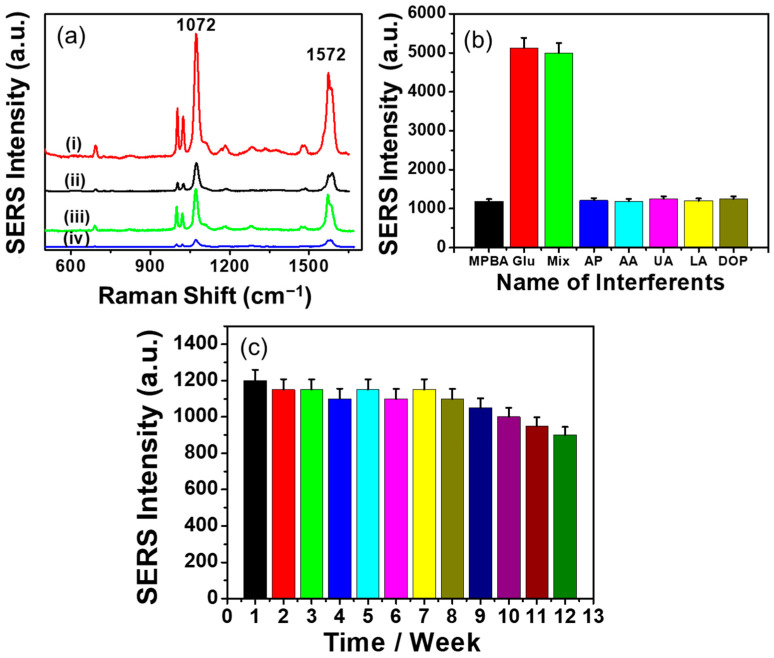
(**a**) SERS spectra of CP/CS/AgNPs/MPBA and CP/AgNPs/MPBA-based platform (i, iii) after and (ii, iv) before 1mM glucose binding. (**b**) Bar graph representation of the SERS responses of other common interfering compounds. (**c**) Bar graph representation of the long-time substrate stability of the CP/CS/AgNPs/MPBA-based SERS platform.

The SERS responses of the CP/CS/AgNPs/MPBA substrate were measured before and after glucose binding and are shown in Figure 4a (black and red line). For a comparison, SERS spectra of a CP/AgNPs/MPBA substrate were also measured before and after glucose binding (blue and green line). The SERS spectra of CP/AgNPs/MPBA and CP/CS/AgNPs/MPBA before glucose binding both show a weak peak at 699 cm^−1^ and four major peaks at 995, 1022, 1072, and 1572 cm^−1^. The weak peak at 699 cm^−1^ is related to the C-C bending and C-S stretching modes [72]. The major peaks at 995 and 1022 cm^−1^ are related to the in-plane benzene ring breathing and are assigned as in-plane C-C-C bending and in-plane C-H bending modes, respectively [72]. The strongest peak at 1072 cm^−1^ arose due to the C-S and B-O stretching modes, whereas the peak at 1572 cm^−1^ is related to the C-C bending modes [72]. The SERS intensities of these peaks in the CP/CS/AgNPs/MPBA substrate are higher than that observed in the CP/AgNPs/MPBA substrate, indicating that the amount of surface-attached MPBA increased in the presence of CS. This is due to the fact that although a sufficient amount of Ag^+^ can be electrostatically adsorbed on CP surface; however, after the growth of nanoparticles on CP surface, many AgNPs detached from the surface as the interaction between the AgNPs and the negatively charged CP surface was weak. On the other hand, when CP was modified with CS with positively charged NH_2_ groups, the surface-formed AgNPs strongly bonded with the NH_2_ group of CS through Ag-N bonding, thus allowing more MPBA on the CP surface through the interaction of Ag-S bonding. After glucose binding, the intensities of the SERS peak in CP/AgNPs/MPBA and CP/CS/AgNPs/MPBA increased due to all vibration modes, caused by the orientation change of MPBA and charge transfer effects [18]. These orientation and charge transfer effects resulted from the binding of glucose with the boronic acid motif, increase the SERS signal of C-S and B-O stretching, and C-C bending in MPBA. However, the SERS intensity of CP/CS/AgNPs/MPBA was found to be about two times higher than that observed in CP/AgNPs/MPBA, indicating a greater amount of MPBA attached to the CP/CS/AgNPs than to the CP/AgNPs surface, resulting in more orientation change of MPBA occurring in CP/CS/AgNPs/MPBA. The intensity of the 1572 cm^−1^ peaks before and after glucose binding also significantly increased; however, the 1072 cm^−1^ peak was considered for analytical purpose due its highest intensity. To assess the selectivity of the present paper-based SERS sensor, other common metabolic compounds such as uric acid (UA), ascorbic acid (AA), dopamine (DA), acetaminophen (AP), and lactic acid (LA) were tested and the bar graph representation of the SERS responses are shown in Figure 4b. The SERS spectra are shown in Figure S4 in the Supporting Information section. The peak at 1072 cm^−1^ did not significantly change when 100-times-higher concentrations of these compounds were treated individually with the substrate. In addition, the ∆SERS response of the mixture of glucose and other interfering compounds also did not significantly increase with that obtained for only glucose. The boronic acid group in MPBA forms a covalent bond with the 1,2-diol group of glucose, creating a boronate cyclic ester. For glucose, the boronate ester reaction occurs at pH 7–8. At this pH, boronate ester reaction may also occur for sucrose and fructose. Thus, sucrose and fructose might interfere with glucose detection. However, UA, AA, AP, and LA are acidic in aqueous solution while dopamine is basic. Thus, no boronate ester reaction occurred at the pH between 7 and 8, giving highly selective detection for glucose against these compounds. These results clearly indicate the high specificity of the present paper-based SERS platform for glucose detection. The long-term substrate stability of the SERS sensor was evaluated by measuring the SERS responses of a glucose-treated single substrate once a week for twelve weeks. The bar graph representation of the SERS responses is given in Figure 4c and the SERS spectra are shown in Figure S5 in the Supporting Information. The SERS responses changed by about 8% after 8 weeks. After 9 and 12 weeks, the responses decreased about 13 and 25%, respectively, indicating that the present paper-based SERS sensor has a long-term substrate stability of 8 weeks.

### 3.5. Optimization of the Experimental Conditions for SERS Measurements

To maximize the SERS response, various experimental parameters such as AgNO_3_, CS, and MPBA concentrations, the pH of the MPBA solution for SAM formation on CP/CS/AgNPs, and the pH of the glucose-binding medium were optimized, with the results shown in Figures S6 and S7. To optimize the maximum growth of the AgNPs on the CP/CS substrate, various concentrations AgNO_3_ were dropped on the CP/CS substrate and reduced with NaBH_4_. Figure S6 showed the SEM images of the AgNPs formed at various AgNO_3_ concentrations ranging between 10 and 100 mM. At low AgNO_3_ concentrations (10 and 20 mM), very few AgNPs were formed and the AgNPs were not distributed uniformly and homogeneously due to the presence of smaller amounts of Ag^+^ ion. At the 50 mM concentration of AgNO_3_, significant amounts of AgNPs were formed, but the distribution are not homogenous and uniform. When the concentration of the AgNO_3_ was 100 mM, the large amount of AgNPs were densely formed and the particles were uniformly and homogeneously distributed. From the SEM images, it is clear that the 100 mM concentration of AgNO_3_ gave the best AgNPs formation in terms of particle number, size, and distribution. Thus, to form the maximum AgNPs on the CP/CS substrate, the AgNO_3_ concentration was optimized at 100 mM. For the optimization of the other conditions, SERS spectra of the glucose detection were measured with CP/CS/AgNPs/MPBA with various CS and MPBA concentrations and pHs of the MPBA solution and glucose-binding medium. The effect of CS concentration was examined between 0 and 3% (Figure S7a). The ∆SERS response gradually increased from 0 to 1% and over 1% concentration, the response did not significantly change due to the saturation effect of the interaction sites in CP. Since CS was attached to the CP surface through the electrostatic interaction between the negatively charged CP and positively charged CS, the CS concentrations at lower concentrations (0.5 and 1%) had enough interaction sites to attach on CP, aiding increasing amount AgNPs and MPBA attachment. As the CS concentrations increased from 1 to 3%, the interaction sites in CP became limited and were not able to attach more CS. Thus, the amounts of AgNPs and MPBA did not significantly change. Thus, the optimum CS concentration was optimized as 1%.

The glucose detection was achieved through monitoring the SERS peak in MPBA. Thus, the effect of MPBA concentration had a big role in glucose detection. The MPBA concentration varied between 1 and 7 mM in the fabrication of CP/CS/AgNPs/MPBA substrate. The ∆SERS responses of the glucose detection were found to be gradually increased from 1 mM to 5 mM and over 5 mM concentration. The ∆SERS response did not significantly change due to the saturation effect of MPBA binding caused by limited amounts of AgNPs. Since AgNPs were grown at the CP/CS surface, we did not attempt to optimize the number of nanoparticles formed. The maximum response was found at the MPBA concentration of 5 mM. Thus the optimum MPBA concentration was considered as 5 mM (Figure S7b).

The ∆SERS responses of the glucose detection were also checked by changing the pH of the MPBA solution for SAM formation on CP/CS/AgNPs (Figure S7c). The orientation of MPBA on metal surface can be varied with pH [50], which had a significant effect on glucose binding. In the present study, the pH of the MPBA in the SAM-forming solution was varied from 3 to 10. At lower pHs of 3 to 6 (acidic condition), the ∆SERS responses of glucose detection were found to be small and did not significantly change. This might be related to the flat orientation of MPBA adsorbed on a metal surface [50]. When pHs of the MPBA-adsorbing solution increased from 7 to 9, the ∆SERS responses rapidly increased and reached a plateau, which might be associated with the perpendicular orientation of the MPBA molecule. Considering the maximum response and glucose detection in the human serum sample at physiological condition, the pH of the MPBA-adsorbing solution was chosen as 7.4.

The optimization of the pH of the glucose-binding medium is important to find out the appropriate body fluids for the sensor’s practical application. Thus, the effect of the pH of the glucose-binding medium was checked in the pH range between 3 and 9. The ∆SERS responses did not significantly change in the pH range between 3 and 5 (Figure S7d). However, over pH 5, the ∆SERS responses rapidly increased up to pH 7.4 and then slightly decreased at pH 9. Since the maximum response was observed at a pH of 7.4, this was selected as the optimum pH of the binding medium. This is also the physiological pH of the blood.

### 3.6. Analytical Evaluation of the Glucose Detection

**Figure 5:**
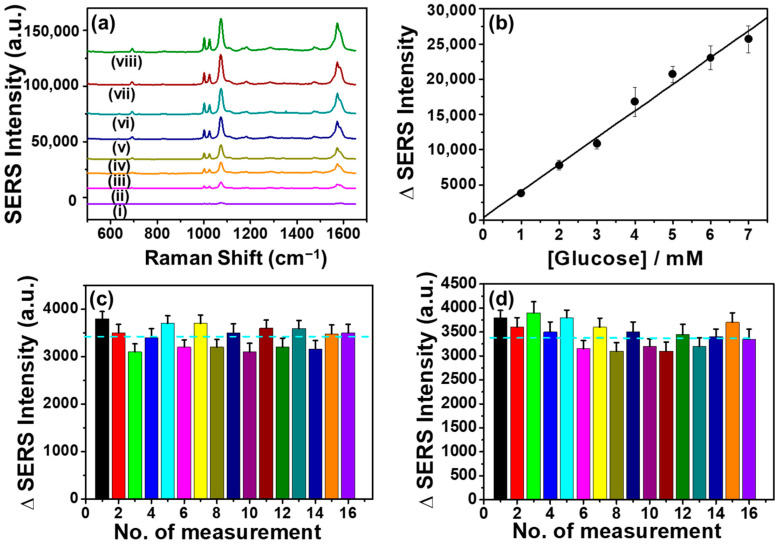
(**a**) SERS spectra of the CP/CS/AgNPs/MPBA-based platform at various glucose concentrations: (i) 0, (ii) 1, (iii) 2, (iv) 3, (v) 4, (vi) 5, (vii) 6, and (viii) 7 mM and (**b**) corresponding calibration plot. (**c**,**d**) Bar graph representation of the SERS responses for (**c**) sixteen different SERS platforms and for (**d**) sixteen random points on a single platform at a glucose concentration of 1 mM.

Under the optimized condition, the CP/CS/AgNPs/MPBA substrate was used for glucose detection. SERS responses were measured at various glucose concentrations ranging from 1 to 8 mM. The SERS intensity linearly increased with the increasing concentration of glucose and was directly proportional to the glucose concentration between 1 and 7 mM as shown in Figure 5a. A calibration plot was constructed by plotting the differences in SERS intensities between the blank and glucose responses as shown in Figure 5b. This linear dependency between differences in SERS intensity (∆I) and the glucose concentrations yielded a regression equation of ∆I = (375.71 ± 932) + (3784 ± 204.35) [C] (mM) with correlation coefficients of 0.992. The CP/CS/AgNPs/MPBA-based SERS platform exhibits a wide linear dynamic range between 1~7 mM. The reproducibility expressed in terms of the relative standard deviation (RSD) was 6.76 (*n* = 5). The limit of detection (LOD) was determined to be 0.74 ± 0.06 mM, which was based on three measurements of the standard deviation of the blank noise (95% confidence level, k = 3, *n* = 5). This LOD value is in the same order of magnitude (sub-mM) as the LOD obtained from a microfluidic paper-based SERS method [59], proving the potential of the present glucose SERS sensor. Additionally, we compared the detection parameters of our glucose sensor with other MPBA-based glucose sensors, with the results presented in Table S1 in the Supplementary Materials. Although others glucose sensors were fabricated based on solid substrates, the LODs and linear dynamic ranges are in the same order of magnitude as our LOD and linear dynamic range. Moreover, none of these reported sensors determined the EF/AEF value and reproducibility of their sensors. Considering the comparable analytical ability and use of a cheap paper-based SERS substrate, the present CP/CS/AgNPs/MPBA-based SERS glucose sensor could be an excellent analytical tool for glucose detection in real biological samples.

### 3.7. Reproducibility

The reproducibility of the CA/CS/AgNPs/MPBA-based SERS glucose sensor was evaluated by measuring the SERS responses of 16 different sensor substrates and 16 random points at a single substrate. Figure 5c,d shows the bar graph representations of the SERS intensities versus the time of measurements and the SERS spectra are given in Figures S8 and S9. When the SERS spectra of 16 different substrates (Figure 5c) were measured, the SERS intensities of all peaks, particularly the peak at 1072 cm^−1^, did not significantly changed, indicating that the fabrication process of the CA/CS/AgNPs/MPBA is reproducible. The ∆I responses of the peak at 1072 cm^−1^ varied from 3100 to 3800 with an average ∆I intensity of 3420.62 ± 231.4. The reproducibility expressed in terms of relative standard deviation (RSD) was determined to be 6.76%, showing the good reproducible surface characteristics of the CP/CS/AgNPs/MPBA substrate. In addition, to further evaluate the reproducibility, the SERS spectra were also measured at 16 random spots of a single substrate and the bar graph representation of the SERS intensity is shown in Figure 5d. The SERS spectra are given in Figure S9 in the Supporting Information section. The average ∆I response was calculated as 3380 ± 255.5, corresponding to an RSD value of 7.6%. The slightly high RSD value might be due to the roughened surface formed upon the repeated laser application in the same substrate. Both RSD values clearly indicated that the CP/CS/AgNPs/MPBA-based glucose sensor exhibited excellent reproducibility. This excellent reproducibility came from the CS modification, facilitating the covalent attachment of enormous AgNPs, creating a stable uniform hot spot configuration of AgNPs.

### 3.8. Real Sample Analysis

**Figure 6:**
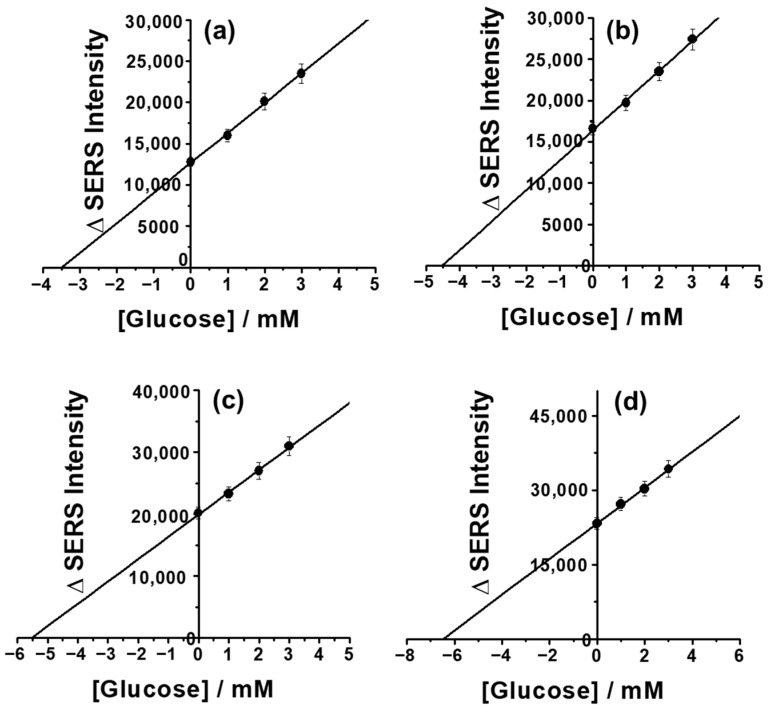
Standard addition plots of the glucose detection in real blood serum samples: (**a**) in a twice-diluted commercial real non-spiked human serum sample and (**b**) 1 mM, (**c**) 2 mM, and (**d**) 3 mM glucose-spiked serum samples.

The applicability of the CP/CS/AgNPs/MPBA-based SERS sensor for glucose detection in commercial real human serum samples was examined. Since the human serum sample might have contained glucose, we first determine the glucose content in serum sample using the standard addition and calibration methods. Figure 6a shows the standard addition curve of a twice-diluted serum sample and the corresponding SERS spectra are given in Figure S10a in the Supporting Information section. The glucose contents in twice-diluted human serum samples was determined as 3.5 ± 0.3 mM. The glucose content was also determined using the calibration method as 3.3 ± 0.45 mM, which is very close to the value determined using the standard addition method. The total concentration of glucose in the human serum sample was determined to be 6.9 ± 0.6. Next, to check the applicability of the SERS platform for glucose detection in real human serum samples, spike and recovery experiments were carried out. Since the serum sample contained 6.9 ± 0.6 mM glucose, it was diluted two times in order to reduce the original glucose content. The twice-diluted serum samples were spiked with 1.0, 2.0, and 3.0 mM of glucose and the quantitative analysis were performed using standard addition methods. Figure 6b–d shows the standard addition plots for the quantification of glucose in glucose-spiked serum samples and the SERS spectra are given in Figure S10b–d in the Supporting Information section. In each case, the SERS responses are linearly proportional to the added concentrations and the extrapolation of the linear line to the negative x axis gives the estimation of the glucose content in serum samples. The recovery results are listed in Table 1, which showed acceptable results with RSD values ranging between 3.4 and 4.5%. The glucose recoveries were between 99 and 101% in spiked human serum samples, which clearly indicated the excellent detection ability of the present CP/CS/AuNPs/MPBA-based SERS glucose sensor in real biological samples.

## 4. Conclusions

A new robust paper-based SERS sensor substrate with enhanced reproducibility, selectivity, and sensitivity was fabricated for the non-enzymatic label-free detection of glucose. The SERS sensor was fabricated by modifying the CP with CS and subsequently depositing AgNPs followed by attaching a glucose-binding receptor, MPBA, on the deposited AgNPs. The glucose detection was carried out by monitoring the C-S and B-O stretching peaks at 1072 cm^−1^ in MPBA, which was found to be increased upon glucose binding due to the orientation and charge transfer effects. The experimental results show that CS modification of the cellulose paper greatly increases the AgNPs amount. This new paper-based SERS sensor can efficiently detect glucose in the presence of other common metabolic compounds without any interference and exhibits a linear dynamic range and LOD of 1~7 mM and 0.74 ± 0.06 mM, respectively. The SERS responses of 1 mM glucose at 16 different substrates and 16 random points at a single substrate yields RSD values of 6.76 and 7.6%, confirming that highly reproducible glucose detection can be achieved. The practical application of this paper-based SERS sensor was tested in non-spiked and glucose-spiked commercial human serum samples. The excellent recoveries of glucose in human serum samples clearly demonstrates the potentiality of the present glucose SERS sensor.

## Figures and Tables

**Table 1:** Spike and recovery results of the glucose detection with CP/CS/AgNPs/MPBA sensor in real commercial human serum samples.

Sample Name	Original Conc. (mM)	Added Conc. (mM)	Found Conc. (mM)	Recovery (%)
Two times diluted human serum	Unknown	0	3.47 ± 0.3 (Standard addition) 3.3 ± 0.45 (Calibration)	
Spike 1	3.47 ± 0.3	1	4.5 ± 0.20	100.7
Spike 2	3.47 ± 0.3	2	5.5 ± 0.35	100.5
Spike 3	3.47 ± 0.3	3	6.4 ± 0.3	98.9

## Data Availability

The original contributions presented in the study are included in the article and Supplementary Materials, further inquiries can be directed to the corresponding author.

## Supplementary Materials

The following supporting information can be downloaded at: https://www.mdpi.com/article/10.3390/bios15030153/s1: Figure S1: AgNPs particle size distribution [1]; Figure S2: XRD diffractograms of CP/CS and CS; Figure S3: SERS and Raman spectra of R6G; Determination of SERS enhancement factor (AEF) of CP/CS/AgNPs substrate estimated from R6G [70]; Figure S4: SERS spectra for the selectivity of the glucose detection; Figure S5: SERS spectra for the long-term stability; Figure S6: SEM images of the optimization of the AgNO_3_ concentration to form AgNPs; Figure S7: Optimization of the various experimental conditions; Figure S8: SERS spectra for the reproducibility experiments at 16 different substrates; Figure S9: SERS spectra for the reproducibility experiments at 16 random points at one substrate; Figure S10: SERS spectra for the glucose detection in the non-spiked and glucose-spiked human serum samples. Table S1: Comparison of the detection parameters with other glucose sensors [17,18,19,20,21,59,73,74].
