# Supplementary material for: A New Chitosan-Modified Paper-Based SERS Glucose Sensor with Enhanced Reproducibility, Stability, and Sensitivity for Non-Enzymatic Label-Free Detection
Source: Biosensors (Basel). 2025 Mar 1;15(3):153. doi: 10.3390/bios15030153 (PMC11940450; doi:10.3390/bios15030153)
Supplement: Supplementary file 1 [file biosensors-15-00153-s001.zip › biosensors-3479022-supplementary.pdf]

Supporting Information

A New Chitosan Modified Paper Based SERS Glucose Sensor with Enhanced Reproducibility, Stability, and Sensitivity for Non-enzymatic Label-free Detection

Rashida Akter,[a] Toeun Kim,[a] Jong Seob Choi,[b] and Hongki Kim[a]*

[a]Department of Chemistry, Kongju National University, Gongju-shi 32588, Republic of Korea,

[b]Division of Advanced Materials Engineering, Kongju National University, Budaedong 275, Seobuk0-gu, Cheonan-si, Chungnam 31080, Republic of Korea

Corresponding author:
Hongki Kim, E-mail: hongkikim@kongju.ac.kr

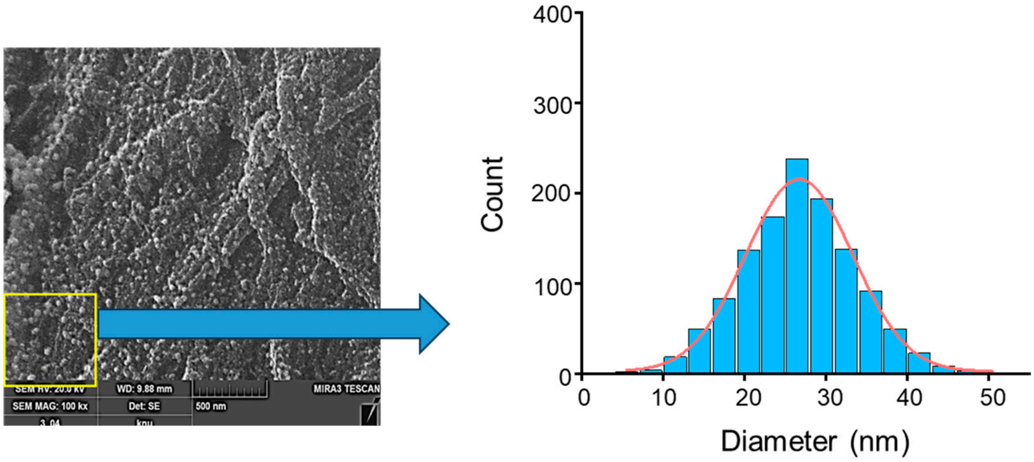

Figure S1. A Gaussian curve for the particle size distribution of AgNPs from the SEM image.

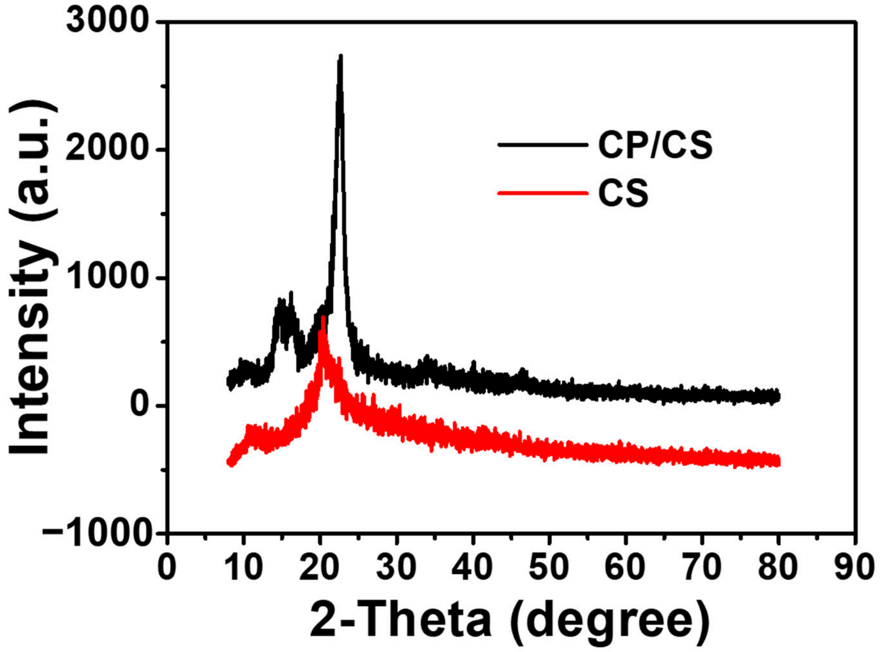

Figure S2. XRD diffractograms of the individual CP/CS and CS.

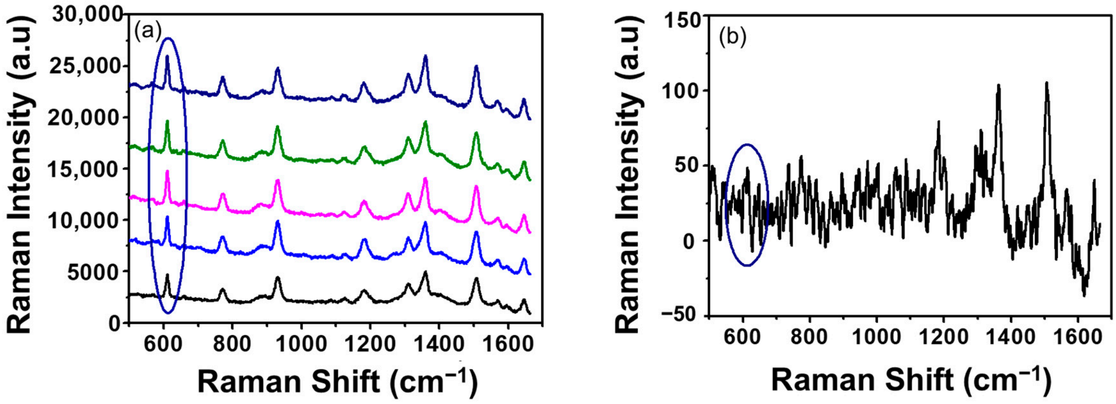

Figure S3. (a) SERS signal of R6G [10^{-6} M] and (b) Raman signal of R6G [2×10^{-1} M].

Determination of Analytical enhancement factor (AEF)

AEF was determined using the following equation [70]:

$$AEF = (I_{SERS}/C_{SERS}) / (I_{RS}/C_{RS})$$

Here, I_{SERS} = 3500 (from Figure S2a)

I_{RS} = 20.51 (from Figure S2b)

C_{SERS} = 1×10^{-6} M

C_{RS} = 2×10^{-1} M

AEF was determined as

$= 3500/1x10^{-6} / 20.51/2x10^{-1}$

$= 3.4 \times 10^{7}$

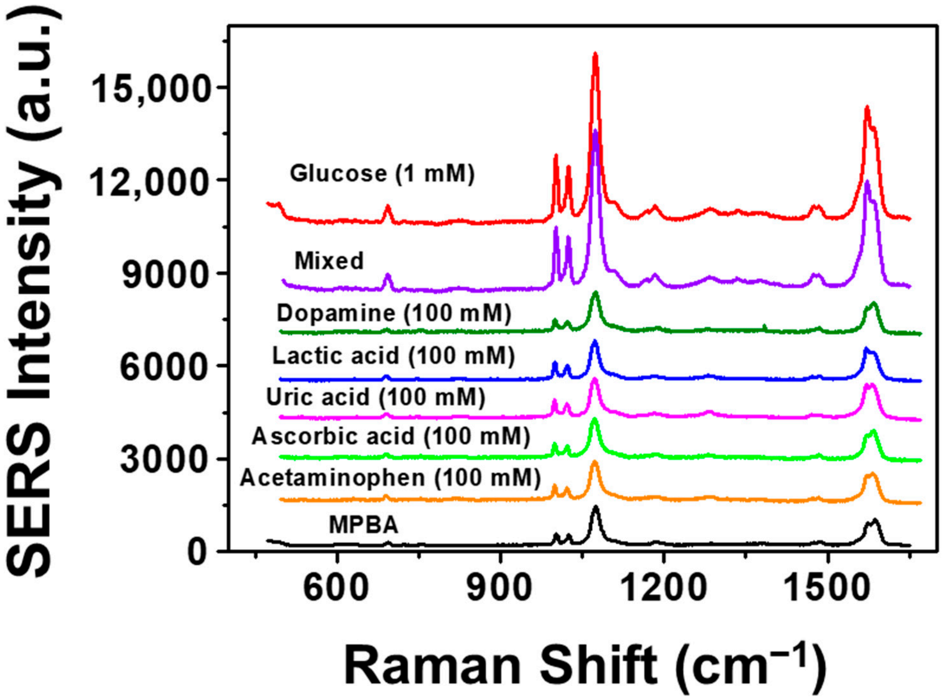

Figure S4. SERS spectra for the selectivity of glucose detection against uric acid, ascorbic acid, acetaminophen, lactic acid, and dopamine.

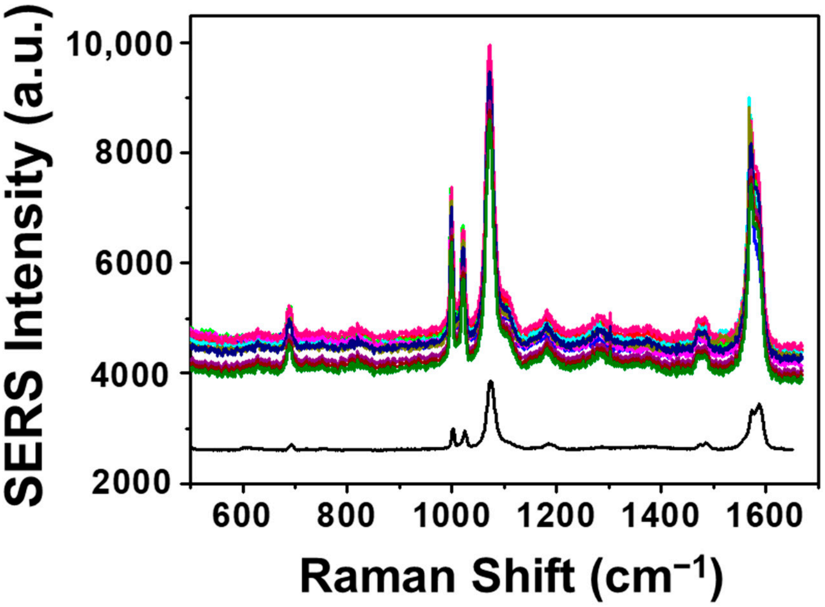

Figure S5. SERS spectra for the long-term substrate stability of the CP/CS/AgNPs/MPBA-based platform.

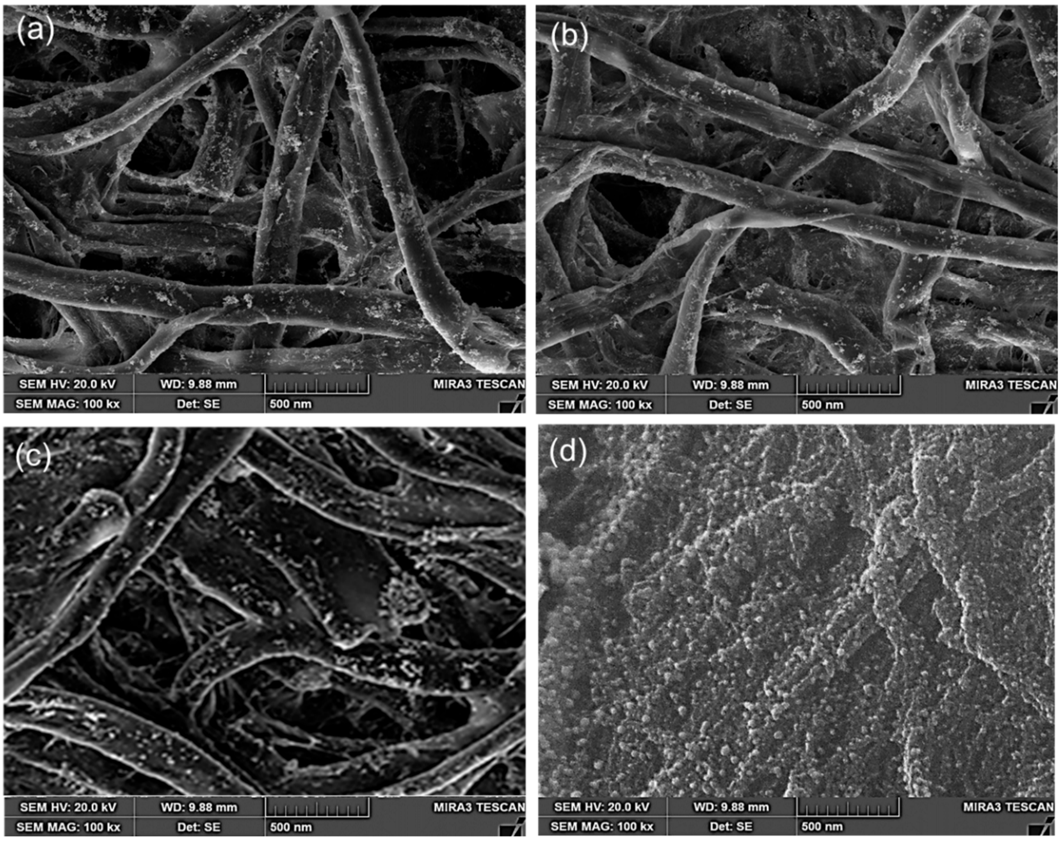

Figure S6. Optimization of the various $AgNO_3$ concentrations to form AgNPs formation. experimental conditions: (a) 10 mM, (b) 20 mM, (c) 50 mM, and (d) 100 mM.

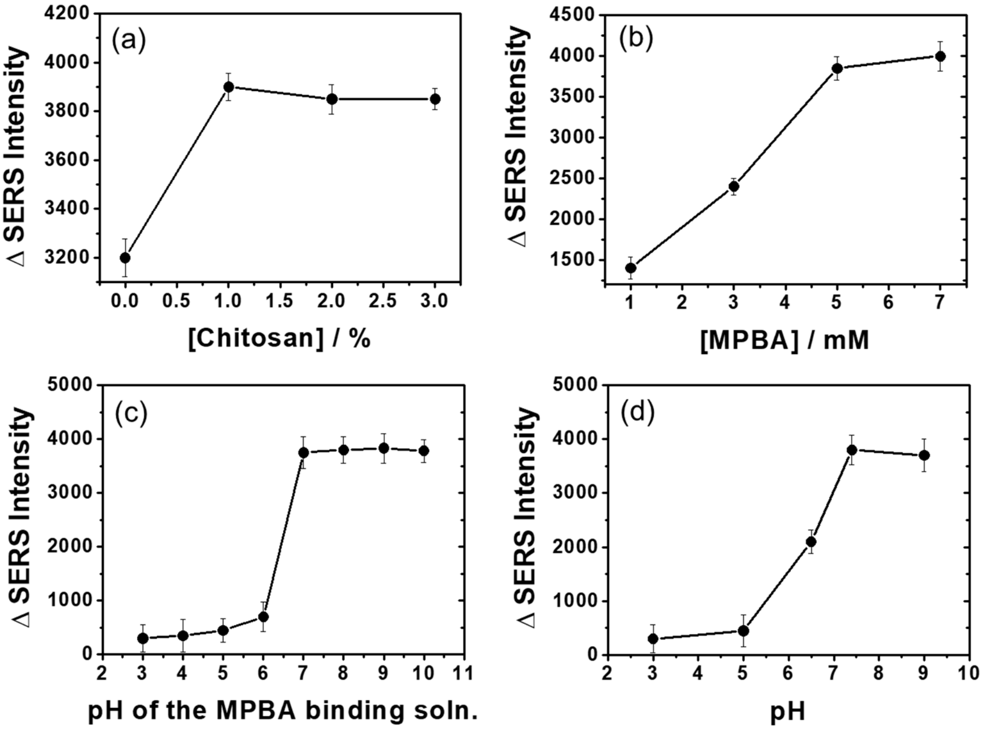

Figure S7. Optimization of the various experimental conditions: (a) CS concentration, (b) MPBA concentration, (c) pH of the MPBA binding solution, and (d) pH of the glucose binding medium.

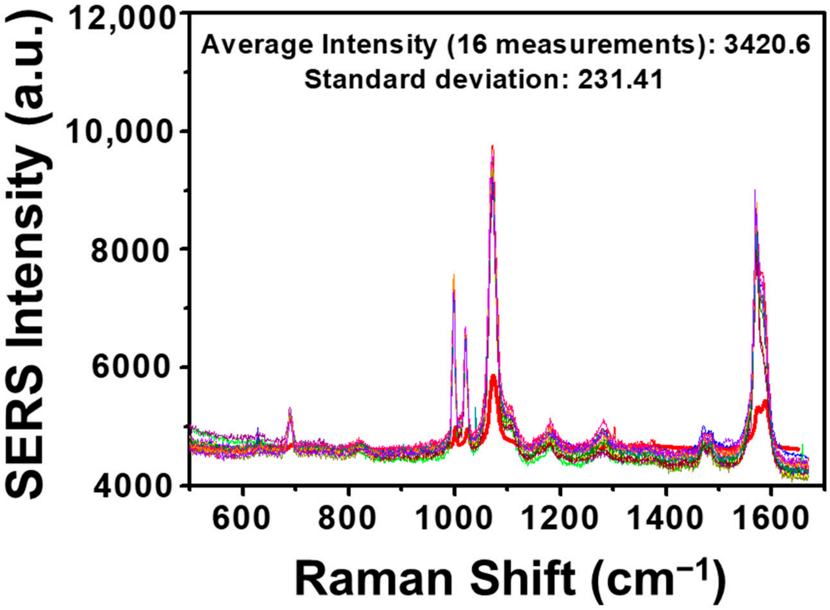

Figure S8. SERS spectra obtained for the 16 different substrates at the glucose concentration of 1.0 mM.

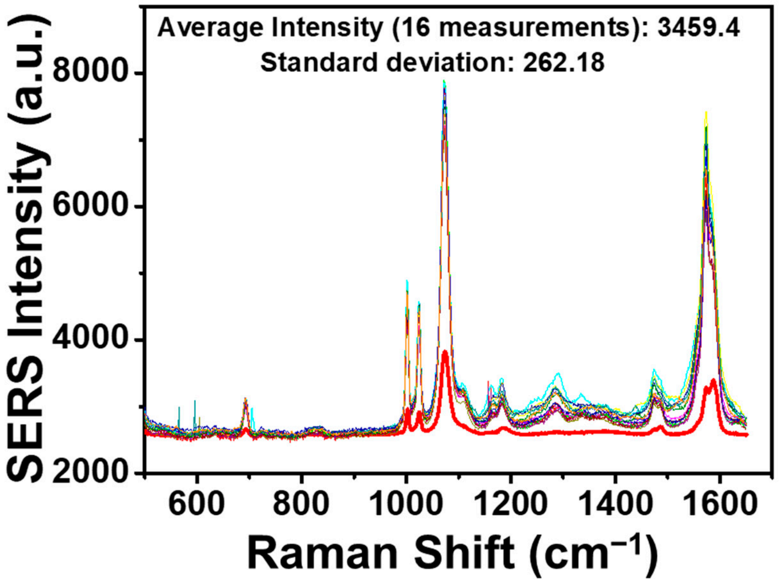

Figure S9. SERS spectra obtained for the 16 random points in a single substrate at the glucose concentration of 1.0 mM.

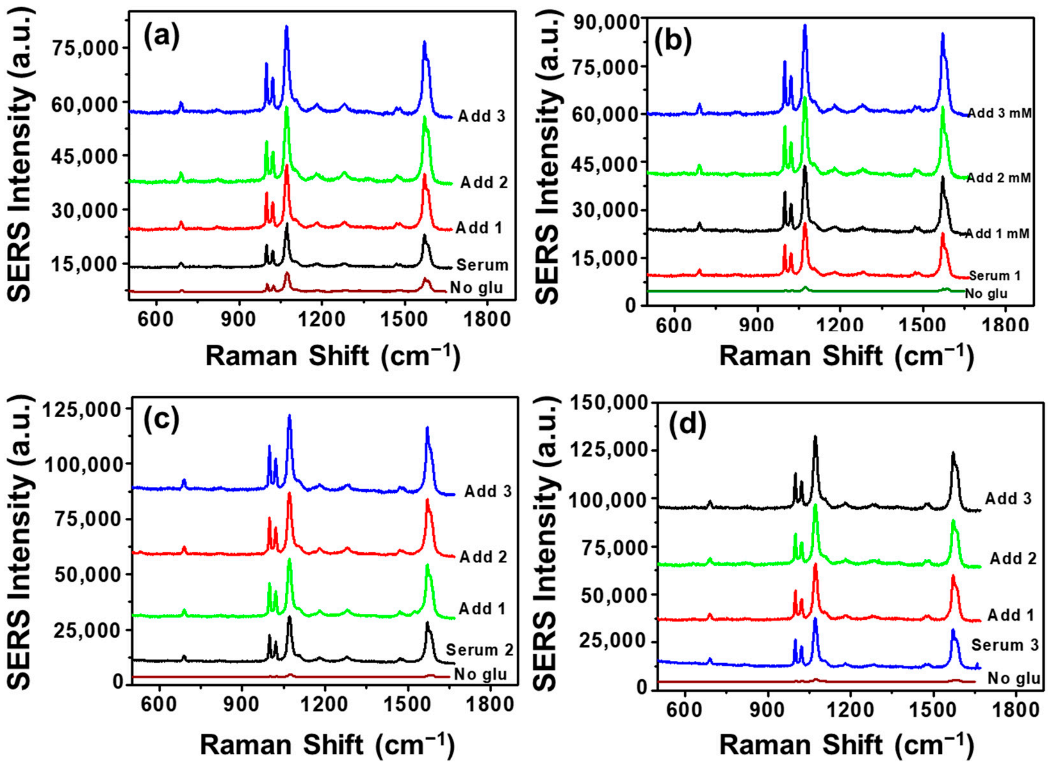

Figure S10. SERS spectra of the glucose detection in non-spikes and glucose-spiked the real human serum samples: (a) human serum, (b) 1 mM, (c) 2 mM, (3) 3mM glucose-spiked serum samples.

]

Table S1. Comparison of detection parameters with other MPBA based glucose sensors.

Substrate	EF/AEF	Linear dynamic range (mM)	LOD (mM)	References
GO@SiO2@AgNPs@4-MPBA		2-20		[2]
		0-20		[3]
Ag nanorod/4-MPBA		2-16	0.5	[4]
Au nanorod/3-MPBA		0-2		[5]
AgNPs/4-MPBA		2-6	0.33	[6]
Ag@AuNPs/GO/4-MPBA		0-25	1.8	[7]
Au nanorod/ 4-MPBA		0.5-10	0.1	[8]
BMFON/4-MPBA		0.1-10	0.1	[9]
CP/CS/AgNPs/4-MPBA	3.4×10^7	1-7	0.74	This work

GO@SiO2@Ag NPs: Silver Nanoparticle-embedded Silica Coated Graphene Oxide; MPBA: 4-mercaptophenyl boronic acid; GO: graphene oxide; AgFON: silver film over nanosphere; BMFON: bimetallic film over nanosphere;

References

1. Langer, J.; Liz-Marzan, L. M. et al. Present and future of surface-enhanced Raman scattering. *ACS Nano* **2020**, *14*, 28-117. doi.org/10.1021/acsnano.9b04224.
2. Pham, X.-H.; Shim, S.; Kim, T.- H.; Hahm, E.; Kim, H.-M.; Rho, W.- Y.; Jeong, D. H.; Lee, Y. S.; Jun, B. – H. Glucose detection using 4-mercaptophenyl boronic acid-incorporated silver nanoparticles-embedded silica-coated graphene oxide as a SERS substrate. *Biochip J.* **2017**, *11*, 46-56. doi.org/10.1007/s13206-016-1107-6.
3. Sun, X.; Stagon, S.; Huang, H.; Chen, J.; Ley, Y. Functionalized aligned silver nanorod arrays for glucose sensing through surface enhanced Raman scattering. *RSC Adv.* **2014**, *4*, 23382-23388. doi.org/ 10.1039/c4ra02423k.
4. Torul H.; Ciftci H.; Dudak F.C.; Adguzel Y.; Kulah H.; Boyac I.H.; Tamer U. Glucose determination based on a two component self-assembled monolayer functionalized surface-enhanced Raman spectroscopy (SERS) probe. *Anal. Methods*. **2014**, *6*, 5097–5104. doi.org/10.1039/C4AY00559G.
5. Li S.; Zhou Q.; Chu W.; Zhao W.; Zheng J. Surface-enhanced Raman scattering behaviour of 4-mercaptophenyl boronic acid on assembled silver nanoparticles. *Phys. Chem. Chem. Phys.* **2015**, *17*, 17638–17645. doi.org/10.1039/C5CP02409A.
6. Gupta, V. K.; Atar, N.; Yola, M. L.; Eryilmaz, M.; Torul, H,; Tamer, U.; Boyaci, I. H.; Ustungag, Z. A novel glucose biosensor platform based on Ag@AuNPs modified graphene oxide nanocomposite and SERS application. *J. Colloid Interface Sci.* **2013**, *406*, 231-237.
7. Shafer-Paltier, K. E.; Hayness, C. L.; Gluksberg, M. R.; Van Duyne, R. P. Toward a glucose biosensor based on surface-enhanced Raman scattering. J. Am. Chem. Soc. 2003, 125, 588-593. doi.org/ 10.1021/ja028255v.

8. Torul, H.; Çiftçi, H.; Çetin, D.; Suludere, Z.; Boyacı, I. H.; Tamer, U. Paper membrane-based SERS platform for the determination of glucose in blood samples. *Anal. Bioanal. Chem*. **2015**, *407*, 8243–8251. doi.org/10.1007/s00216-015-8966-x.
9. Kong, K. V.; Lam, Z.; Lau, W. K. O.; Leong, W. K.; Olivo, M. A transition metal carbonyl probe for use in a highly specific and sensitive SERS-based assay for glucose. J. Am. Chem. Soc. 2013, 135. 18028-18031. doi.org/10.1021/ja409230g
